# Tolerability, acceptability, and reproducibility of topical STAR particles in human subjects

**DOI:** 10.1002/btm2.10524

**Published:** 2023-04-18

**Authors:** Youngeun Kim, Jae Hwan Jung, Andrew R. Tadros, Mark R. Prausnitz

**Affiliations:** ^1^ School of Chemical and Biomolecular Engineering Georgia Institute of Technology Atlanta Georgia USA; ^2^ Department of Pharmaceutical Engineering Dankook University Cheonan Republic of Korea

**Keywords:** dermatology, microneedle, STAR particle, tolerability, topical drug delivery

## Abstract

Topical delivery to treat dermatological disease is constrained by low skin permeability to most drugs due to the stratum corneum barrier. STAR particles containing microneedle protrusions can be topically applied on the skin to create micropores that dramatically increase skin permeability, even to water‐soluble compounds and macromolecules. This study addresses the tolerability, acceptability, and reproducibility of STAR particles rubbed on the skin at multiple pressures and after multiple applications to human subjects. One‐time STAR particle application at pressures between 40 and 80 kPa showed that skin microporation and erythema directly correlated with increased pressure, and 83% of subjects reported STAR particles to be comfortable at all pressures. Repeated application of STAR particles for 10 consecutive days at 80 kPa showed that skin microporation (~0.5% of skin area), erythema (low‐to‐moderate), and comfort with self‐administration (75%) were similar over the course of the study. Comfort of sensations associated with STAR particles increased from 58% to 71% during the study, and familiarity with STAR particles increased from 12.5% to 50% of subjects reporting STAR particle application not feeling different from other skin products. This study demonstrates that topically applied STAR particles were well tolerated and highly acceptable after application at various pressures and repeated daily use. These findings further suggest that STAR particles offer a safe and reliable platform to enhance cutaneous drug delivery.

## 1 INTRODUCTION

Dermatological disorders are the fourth most common cause of nonfatal disease globally, affecting about one‐third of the world population at a given time.^1^, ^2^, ^3^ Patients suffer not only from the conditions of the skin disease itself, but also from the associated psychological and economic burden.^4^, ^5^ In addition, skin diseases cause significant morbidity and mortality to patients in low‐resource settings who may have limited access to treatments or healthcare providers.^6^ Hence, dermatological diseases are of significant public health concern,^2^ and there is a need for efficacious therapy to treat various dermatological conditions.

Pharmacologic treatment of dermatological disease is challenging, as very few drugs have the physicochemical properties required to cross the skin's barrier for successful topical therapy.^7^ Skin is an excellent barrier to absorption of exogenous molecules due largely to its outermost layer called stratum corneum that is typically 10–20 μm thick.^8^ The stratum corneum is composed of keratin‐rich corneocytes surrounded by an extracellular matrix of lipids. There are very few drugs that are sufficiently small (<500 Da) and lipophilic to cross the stratum corneum to achieve therapeutic drug levels in the skin.^9^ Hence, most topical therapies that are typically applied to the skin as a liquid, semisolid, or adhesive patch have poor cutaneous bioavailability that limits effective treatment.^10^, ^11^

Besides topical therapies, skin diseases can also be treated by administering drugs systemically by the oral route or via injection. However, systemic therapies can be limited by side effects due to non‐targeted delivery to the skin and cumulative toxicity risks when drugs are administered long‐term (e.g., hepatotoxicity, nausea).^12^ Local delivery to the skin can be achieved by intralesional injections in the localized skin area (e.g., steroid injections for keloid scars).^13^, ^14^ However, injections require trained health care personnel, and are painful and invasive.

Bioavailability of topical drugs can be improved by increasing skin permeability using chemical or physical methods. Chemical methods improve drug absorption by increasing stratum corneum permeability via chemical enhancers (e.g., alcohols and glycols) added to the drug formulation.^15^, ^16^, ^17^ However, many chemical enhancers irritate the skin and are only moderately effective to increase delivery of small molecules. Physical methods, such as iontophoresis,^18^ electroporation,^19^ and thermal ablation,^20^ involve the use of external energy to breach the skin barrier to increase skin permeability. However, these techniques are often costly, complex, and limited in utility, as they can only be used on small areas (e.g., centimeter‐scale) of the skin.^21^

Microneedles provide a simple, low‐cost method of penetrating the skin barrier for delivery of pharmaceuticals—including water‐soluble drugs, proteins, vaccines, DNA/RNA, and other compounds—in a minimally invasive manner.^22^, ^23^, ^24^, ^25^ Usually in a patch‐based format, microneedles are sub‐millimeter solid needles that can contain encapsulated or coated drugs for direct deposition in the skin. They can also be used via a “poke‐and‐patch” method to create micron‐scale pores in the skin that increase skin permeability to drugs in a topical formulation applied to the treated site. For cosmetic application, microneedles have been incorporated onto the surface of cylindrical rollers (e.g., Dermaroller) to similarly pretreat the skin with micropores to increase dermal absorption.^26^

These microneedle‐based methods have limitations for dermatological treatments, in part because many dermatological indications cover large skin areas (e.g., hundreds or thousands of square centimeters). In addition, many cutaneous diseases can be nonhomogeneously distributed across the body (e.g., vitiligo, psoriasis) which would further complicate patch‐based approaches. Microneedle patches are most suitable for treating areas of a few square centimeters, since larger patches can be difficult to administer and applying multiple patches to cover large or distributed areas can be cumbersome.^22^, ^23^, ^24^, ^25^ Microneedle rollers can be applied over large areas, but require a two‐step process of first using a special device to create the micropores and then applying a topical formulation to the treated site.^26^

To overcome these limitations of microneedles, we developed STAR particles, which are millimeter‐scale particles with micron‐scale projections (i.e., microneedles) made of biocompatible materials (e.g., titanium dioxide).^27^ After incorporation of STAR particles into a topical formulation, the conventional rubbing action associated with applying a topical formulation is sufficient to disrupt the skin's stratum corneum barrier in a minimally invasive, single‐step, and intuitive manner. In this way, a drug can be administered topically for targeted delivery to the skin using a method that is simple and familiar to patients, while also being effective for a broad range of drugs that would normally not be bioavailable via the skin. Topical formulations containing STAR particles can also be applied to large and variable skin areas to treat various dermatological indications distributed across the body.

Previous studies have demonstrated that STAR particles can increase drug and vaccine delivery into skin models ex vivo and rodent skin in vivo, and were well tolerated by human subjects after a single application at a specified application pressure.^27^ However, skin therapies typically require repeated (e.g., daily) applications of topical formulations,^28^ and the pressure applied by a patient may vary. Hence, the effects of repeatedly applying STAR particles to skin, as well as applying STAR particles at different pressures, needs further evaluation.

In this study, we investigated the tolerability, acceptability and reproducibility of STAR particles when applied at three different pressures and when applied repeatedly for 10 consecutive days to the skin of human subjects. In our first study, we used STAR particles at three different application pressures between 40 and 80 kPa to evaluate tolerability and acceptability. In the second study, we applied STAR particles at the highest application pressure (80 kPa) for 10 consecutive days to evaluate safety, tolerability, acceptability, and reproducibility.

## 2 MATERIALS AND METHODS

### 2.1 Manufacturing of STAR particles

STAR particles were manufactured using CO_2_ laser ablation (VLS3.50; Universal Laser Systems, Scottsdale, AZ) from 100 μm thick titanium dioxide green tape (Maryland Ceramic & Steatite, Bel Air, MD), as described previously.^27^ The tape was cut with the following settings: 15% power, 100% speed, and 1000 pulses per inch. After cutting, the particles were washed with DI water and dried in a vacuum oven at 40°C for 8 h. Then, the particles were placed on magnesium oxide trays (Alfa Aesar, Haverhill, MA) and sintered in a furnace (RHF 16/3; Carbolite Gero, Sheffield, United Kingdom) with the following temperature cycle: ramp from room temperature (20–25°C) to 600°C at 2°C min^−1^, hold at 600°C for 1 h, ramp at 5°C min^−1^ to 1000°C, hold at 1000°C for 4 h, and finally cool to room temperature at 10°C min^−1^. After sintering, STAR particles were examined using scanning electron microscopy (TM3000 scanning electron microscope; Hitachi, Tokyo, Japan). After manufacturing, STAR particles were mixed with aloe gel (Fruit of the Earth, Fort Worth, TX) at a concentration of 10 wt%. Low bioburden of STAR particle formulations for human studies was verified (STERIS Laboratories, Mentor, OH) before application to human subjects.

### 2.2 Ex vivo skin treatment with STAR particles

Skin was obtained from excised porcine ears (Pel‐Freeze Biologicals, Rogers, AR) after careful dissection from the underlying cartilage. After removing hair from the skin by shaving (Dyanarex, Orangeburg, NY), the skin was wrapped in aluminum foil and stored at −80°C until further use.

To apply STAR particle formulation on ex vivo skin, skin was thawed in a room‐temperature water bath. For skin treatment studies with varying application pressure, 10 wt% STAR particles suspended in 0.3 g aloe gel were applied to 6.5 cm^2^ of skin. The gel formulation was rubbed onto the skin using light, medium, and high pressure (approximately 40, 60, 80 kPa, respectively) with gloved fingers (Microflex Powder‐Free Nitrile gloves; VWR, Radnor, PA) in a circular motion for 10 s. The application pressure was monitored in real‐time with a force sensor (DI‐1000; Loadstar Sensors, Fremont, CA), that was placed below the skin. After application, the gel formulation was wiped away using isopropyl alcohol wipes (BD, Franklin Lakes, NJ).

### 2.3 Skin assessment after STAR particle treatment ex vivo

After STAR particle treatment, gentian violet (Humco, Austin, TX) was applied to treated skin sites to stain micropores made in the skin by the STAR particles. After ~10 min, the staining solution was removed with isopropyl alcohol wipes (BD) and images of the skin were taken using a stereoscopic microscope (SZX16; Olympus, Tokyo, Japan).

### 2.4 Evaluation of STAR particle treatment in human subjects

STAR particles were applied to human participants with written informed consent to evaluate the tolerability, acceptability, and reproducibility of STAR particle application, as approved by Georgia Tech's Institutional Review Board (IRB #H16410). Potential participants recruited in the study were excluded if they had diseased or abnormal skin at the STAR particle application sites, were using any medicine on the application sites, had a disease or condition that affects pain sensation, or had an allergy to materials that were used to make STAR particles, aloe gel, or gentian violet. Demographic information of the subjects is included in Table S1.

For the first study, in which STAR particles were applied at variable pressures, six subjects were recruited. For the second study, in which STAR particles were applied for 10 consecutive days at high pressure, six subjects from the first study and two additional subjects were recruited. In both studies, STAR particles formulated in aloe gel (1 g total, 10 wt% STAR particles) were applied on the anterior forearm of each subject. Before application of a formulation, the skin surface was cleaned with alcohol wipes and let dry for 1 min. In the first study, the area of each application site was 6.5 cm^2^, and the sites were spaced 2.5 cm apart, edge‐to‐edge. In the second study, the area of each application site was 26 cm^2^ and the sites were spaced 5 cm apart, edge‐to‐edge. Aloe gel for the control sites or STAR particle formulation for the treatment sites was applied in a circular motion for 10 s with gloved fingers and was subsequently thoroughly removed with cotton pads (Crosstex, Hauppauge, NY) that were moistened with sterile water (Corning, Glendale, AZ). Study participants were blinded during the aloe gel and STAR particle application, and aloe gel and STAR particle formulations were applied in a randomized order. Additionally, all skin procedures were done by the same investigator to ensure consistent treatment.

### 2.5 Assessment of skin application sites

Immediately after each time STAR particles were applied, the skin was scored for erythema size and intensity using a scale shown in Table S2. The skin was also stained with gentian violet, and stereoscopic images of the skin were taken. The images were then processed with ImageJ (National Institutes of Health, Bethesda, MD) to evaluate skin puncture sites as previously described.^27^ Participants were also asked to complete a short questionnaire to report their sensations during the STAR particle applications and describe their experiences.

### 2.6 Statistical analysis

Statistics were calculated using Prism software version 9.1.1 (GraphPad, San Diego, CA). A Student's *t*‐test or one‐way ANOVA was used to determine statistical significance when comparing two or more datasets, respectively. *p* Values of <0.05 were considered statistically significant.

## 3 RESULTS

### 3.1 Preparation and characterization of STAR particles

**FIGURE 1:**
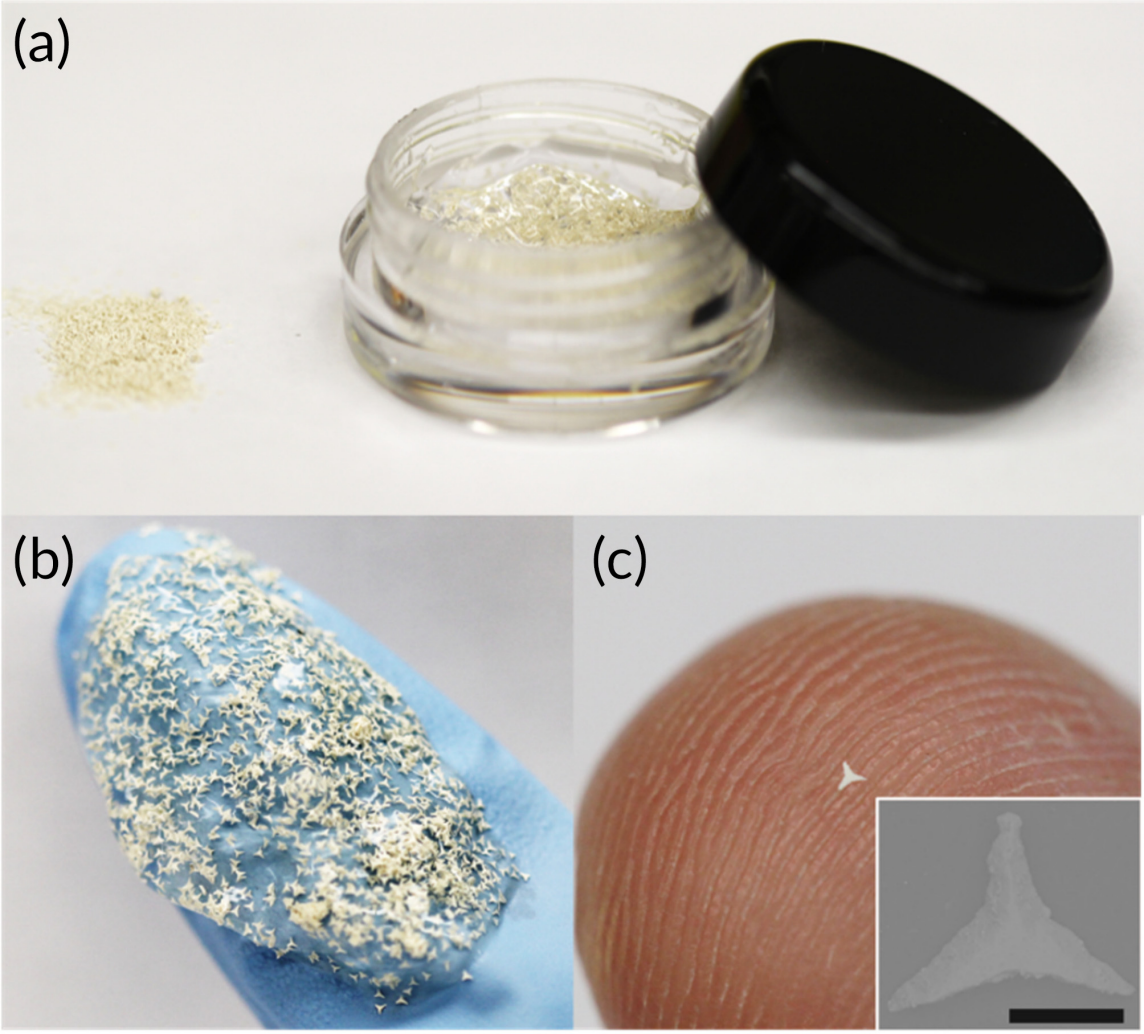
STAR particles contain micron‐scale projections designed to create micropores in the skin. (a) Representative photographic image of a dose of STAR particle formulation in dry form (left) and a plastic container with aloe gel (right). (b) Representative photographic image of STAR particle formulation distributed on a gloved finger. (c) Representative photographic image of an individual STAR particle on a fingertip; inset shows a representative scanning electron microscopy image of a STAR particle (scale bar = 250 μm).

Fabrication of STAR particles was conducted as previously described.^27^ Briefly, we laser cut and sintered titanium dioxide sheets to produce STAR particles, which were then formulated in aloe gel (Figure 1a) for manual application to the skin (Figure 1b). Each STAR particle had three microprojection arms, measuring 310 ± 14 μm in length with a tip‐to‐tip distance of 551 ± 29 μm and tapering to a tip with a radius of 16 ± 4 μm (mean ± standard deviation, *n* = 4 replicates; Figure 1c).

These STAR particles were first tested on porcine skin ex vivo to verify their functionality to create micropores in the skin before conducting the study on human subjects. After rubbing STAR particles onto porcine skin using low (~40 kPa), medium (~60 kPa), high (~80 kPa) pressure, gentian violet staining of the skin revealed a characteristic “freckled pattern” of micropores, which indicated successful skin micropuncture by the STAR particles at all three application pressures (Figure S1).

### 3.2 Effect of STAR particle application pressure in human subjects

After confirming STAR particle functionality ex vivo, we carried out a study in six human subjects to determine the effect of STAR particle application pressure on (i) creation of micropores in human skin and (ii) tolerability and acceptability reported by the subjects. We tested the effect of application pressure to assess the impact of possible user‐to‐user variability when patients might apply STAR particles themselves (although in this study STAR particle formulations were applied by a single study investigator). In addition to applying STAR particle formulations at various pressures, we also applied aloe gel without STAR particles as a negative control and inserted a 26‐gauge hypodermic needle to a depth of 0.5 cm into the skin as a positive control.

#### 3.2.1 Skin micropuncture

**FIGURE 2:**
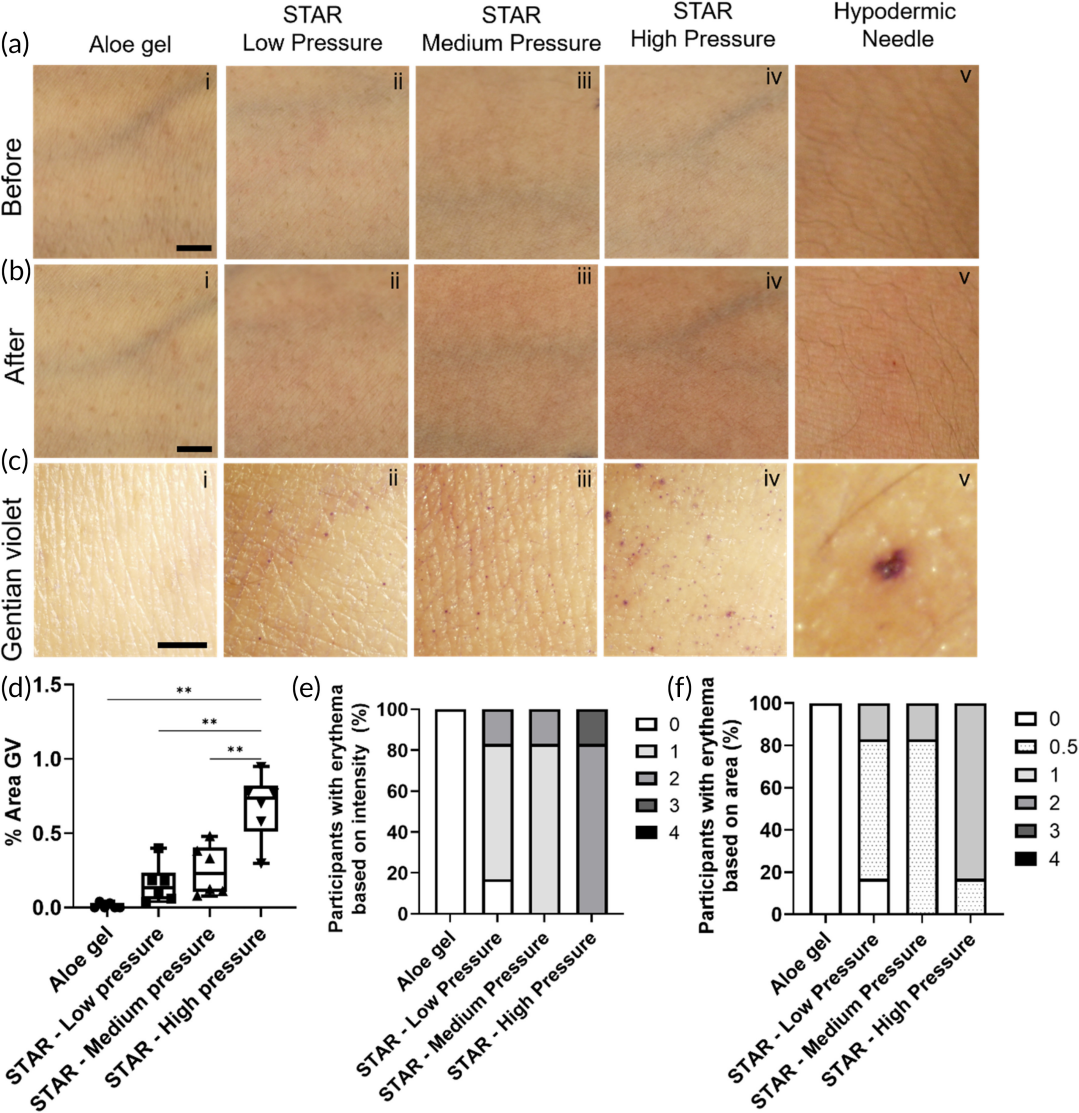
Tolerability and efficacy of STAR particles applied to human subjects at different pressures. Representative photographic images of skin before (a) and after (b) treatment with: aloe gel (without STAR particles) at high pressure (80 kPa), STAR particles at low application pressure (40 kPa), STAR particles at medium application pressure (60 kPa), STAR particles at high application pressure (80 kPa), or a hypodermic needle (scale bar = 5 mm). (c) Representative brightfield microscopy images of skin after gentian violet staining to visualize possible micropores that resulted from each treatment (scale bar = 2 mm). (d) Quantification of gentian violet skin staining as a percent of treatment area. Data are shown as individual data points as well as in a box‐and‐whisker format based on six replicates. Statistical comparisons are based on a one‐way ANOVA: **p < 0.05. Analysis of erythema after skin treatments in terms of intensity (e) and area (f) of erythema. Data were collected from six human subjects. Scoring rubric shown in the key can be found in Table S2 and average erythema scores can be found in Table S3.

We first assessed the effect of application pressure on STAR particle puncture of the skin in human subjects. Gentian violet staining showed sites of skin micropuncture after STAR particle treatment, with more micropores evident at higher application pressure (Figure 2c, ii–iv). A larger site of skin puncture was seen at the site of hypodermic needle application (Figure 2c, v) and no evidence of skin puncture was seen after application of gel without STAR particles (Figure 2c, i).

To quantify the extent of skin micropuncture, we determined the total area of gentian violet staining and found that it increased with increasing application pressure (ANOVA, *p* < 0.05) and was always higher than the gel‐only control (Student's *t*‐test, *p* < 0.05) (Figure 2d). This indicates that the STAR particles created micropores across stratum corneum and the extent of microporation increased with application pressure.

#### 3.2.2 Tolerability

We next assessed erythema. We took images of the skin before (Figure 2a, i–v) and after each application and generally observed mild erythema after application of STAR particles at all three pressures, as well as after application of the hypodermic needle (Figure 2b, ii–v). In contrast, no erythema was observed on skin sites with gel that did not contain STAR particles (Figure 2b, i).

We quantified the erythema and found that intensity (Figure 2e) and area (Figure 2f) of erythema were similar after application of STAR particles at low or medium pressure, mostly having an intensity score of 1 (i.e., very slight erythema, barely perceptible) and an area score of 0.5 (i.e., not spreading beyond the area of STAR particle application). STAR particle application at high pressure generally had an intensity score of 2 (i.e., well‐defined erythema) and an area score of 1 (i.e., erythema mildly spreading beyond application site). Hypodermic needle insertion also usually yielded erythema intensity and area scores of 2 and 1, respectively, while gel application without STAR particles produced erythema scores of zero (Figure 2e,f). Average erythema scores are shown in Table S3. Other than erythema, no edema, tenderness, lasting pain or other adverse effects were seen, other than a spot of blood that was sometimes present after hypodermic needle insertion.

#### 3.2.3 Acceptability

**FIGURE 3:**
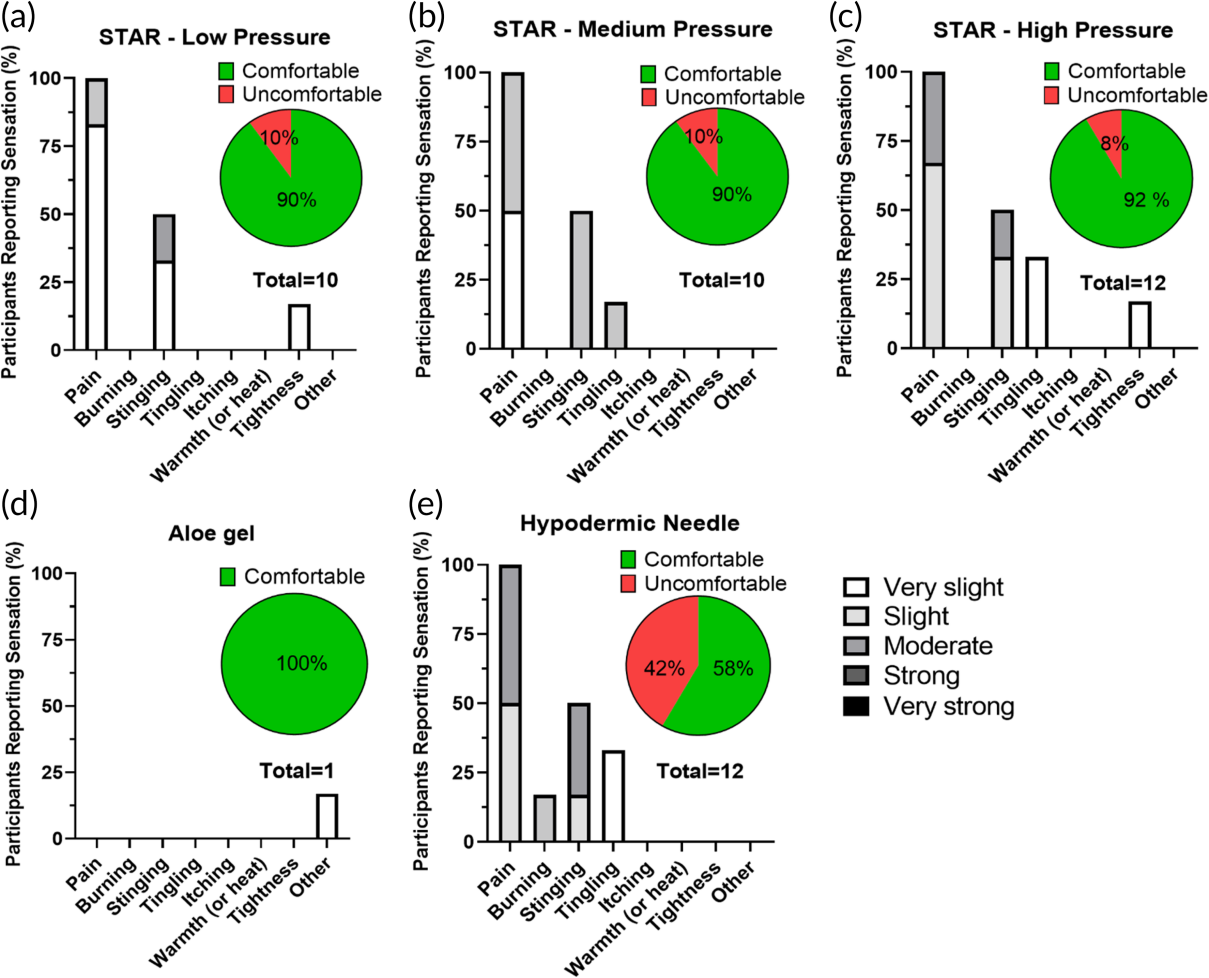
Sensations reported after application of STAR particles to human subjects at different pressures. Frequency that different types of sensations were reported from: (a) STAR particle application at low pressure (40 kPa), (b) STAR particle application at medium pressure (60 kPa), (c) STAR particle application at high pressure (80 kPa), (d) aloe gel application (without STAR particles) at high pressure (80 kPa), and (e) hypodermic needle application. Insets show percentage of sensations reported to be comfortable. The key on the lower right shows degree of sensation reported by participants. Data were collected from six human subjects.

Considering acceptability of STAR particles, study participants all reported some level of sensation associated with STAR particle application. Five of the subjects (83%) reported the sensations to be comfortable, while one subject (17%) reported discomfort, independent of the pressure of application (Figure 3a–c). Subjects assigned one or more descriptions to the sensations they felt, with pain being reported by 100% of subjects, stinging by 50% of the subjects followed by tingling and tightness. The only sensation reported to be uncomfortable was pain (i.e., by one subject). The intensity of the sensations was generally very slight during low pressure application; very slight or slight during medium pressure application; and very slight, slight or moderate during high pressure application.

Gel without STAR particles was uniformly reported as comfortable and produced essentially no reported sensation (Figure 3d). The hypodermic needle was reported as comfortable by four subjects (67%) and as uncomfortable by two subjects (33%) (Figure 3e). All subjects reported pain that was slight to moderate and some subjects reported stinging, tingling and/or burning that were very slight, slight or moderate. From this, we conclude that STAR particles, even when applied at the highest pressure, were generally accepted and tolerated well by the study subjects.

### 3.3 Effect of repeated application of STAR particles in human subjects

Because most dermatological treatments require multiple applications, we carried out an additional study in which STAR particles were applied for 10 consecutive days to eight human subjects (i.e., the same six subjects from the first study, plus two more). To provide a rigorous assessment of the effect of repeated application, we administered the STAR particles at high pressure (80 kPa) and at the same skin site every day. All studies were carried out with informed consent by participants and with approval by the Georgia Tech IRB.

#### 3.3.1 Skin micropuncture

**FIGURE 4:**
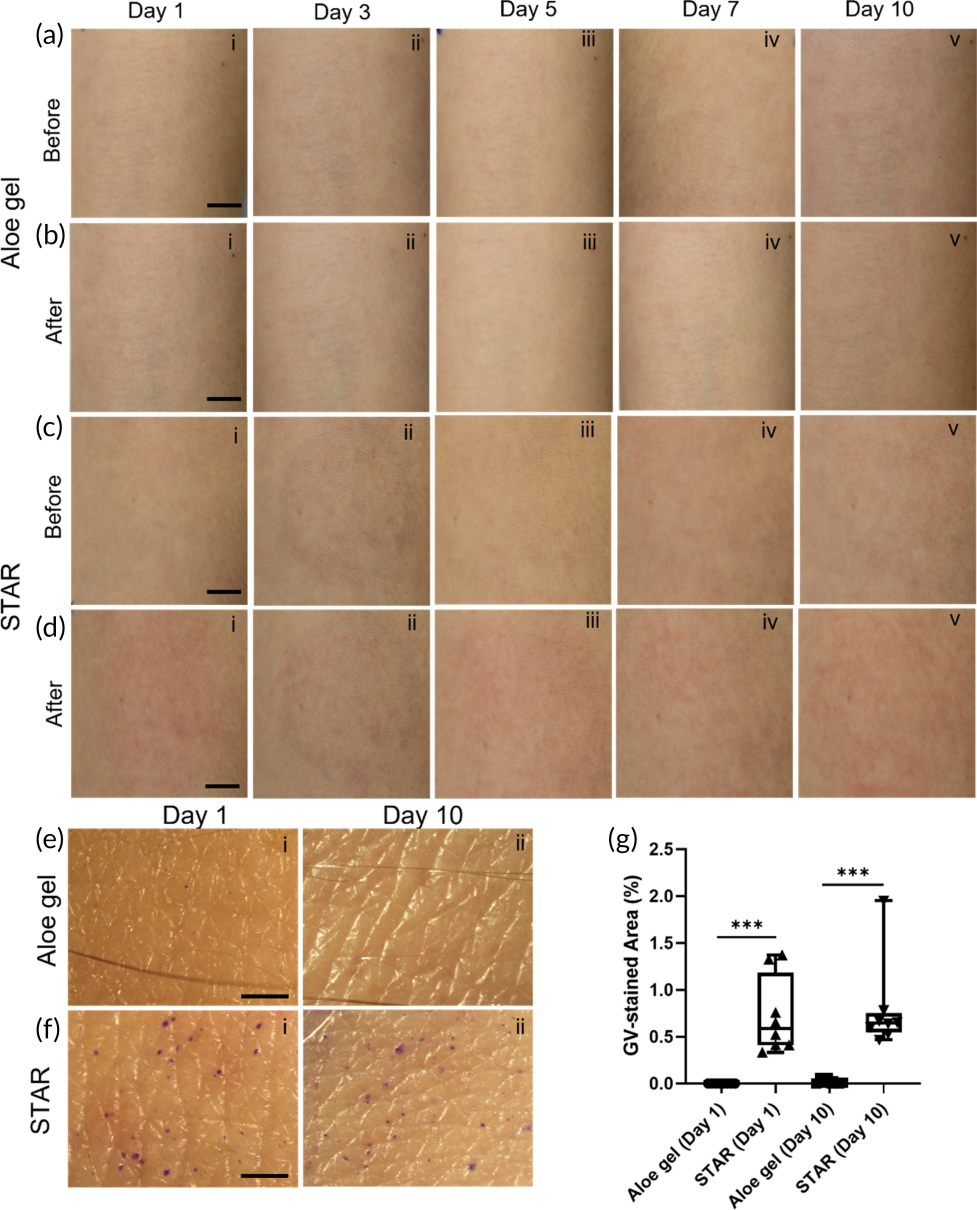
Tolerability and efficacy of daily application of STAR particles in human subjects for 10 consecutive days. Representative photographic images of skin before (a) and after (b) applying aloe gel (without STAR particles) at high pressure (80 kPa) and before (c) and after (d) applying STAR particles at high pressure (80 kPa) over the course of daily skin treatment (scale bars = 1 cm). Representative brightfield microscopy images of skin after gentian violet staining to visualize micropores that resulted from aloe gel (e) and STAR particle (f) treatment on Day 1 and Day 10 of the study (scale bar = 1 mm). (g) Quantification of gentian violet skin staining as a percent of treatment area. Data are shown as individual data points as well as in a box‐and‐whisker format based on eight replicates. Statistical comparisons are based on Student's *t*‐test: ***p < 0.001.

Like the first study, we observed micropores in the skin after STAR particle application (Figure 4f), but not after rubbing with gel containing no STAR particles (Figure 4e). Qualitative (Figure 4e,f) and quantitative (Figure 4g) assessments showed that skin microporation did not significantly change between STAR‐treated sites on Day 1 and Day 10 (Student's *t*‐test, *p* > 0.05; Figure 4g). This suggests that micropores did not accumulate over time and that the skin did not become more or less susceptible to microporation after repeated use of STAR particles.

#### 3.3.2 Tolerability

**FIGURE 5:**
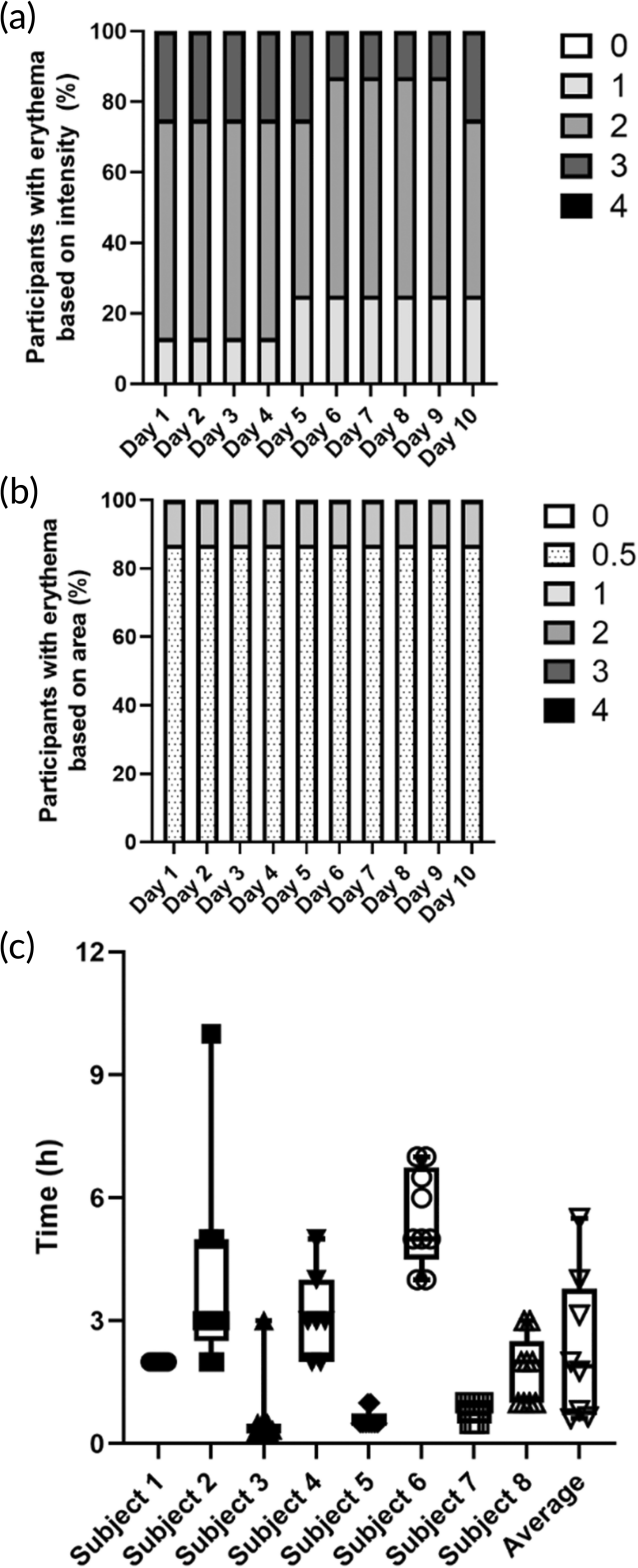
Erythema from daily application of STAR particles in human subjects for 10 consecutive days. (a) Erythema intensity. (b) Erythema area. (c) Self‐reported duration of erythema from the study participants for each of the 10 applications shown with individual data points as well as in a box‐and‐whisker format. STAR particles were applied at high pressure (80 kPa). Data were collected from eight human subjects. Scoring rubric shown in the key can be found in Table S2 and time course data corresponding to each individual subject in (c) is shown in Figure S2.

We again found evidence of erythema in the skin after STAR particle application (Figure 4c,d) and not after application of gel without STAR particles (Figure 4a,b). Over time, erythema intensity and area showed little change during the 10‐day study (Figures 4c,d and 5a,b). This indicates that the skin was neither sensitized nor desensitized to STAR particle treatment over the course of 10 days.

Subjects self‐reported the time for erythema to disappear as 2.3 ± 1.8 h (mean ± standard deviation; Figure 5c). For a few subjects, there was notable fluctuation in their reported times, as some of them stated that they had forgotten to monitor their arms for a few hours following the visit, which suggests that the time for erythema to disappear may have been shorter than reported. When erythema disappearance time was plotted as a function of time for each subject, there was no consistent trend of increasing or decreasing time observed (Figure S2), which is consistent with the observation that erythema intensity and area did not significantly change over the course of the study either (Figure 5a,b).

Other than erythema, there was just one adverse event throughout the 10‐day study. One subject had mild skin irritation that looked like a rash at the site of STAR particle application that appeared on Day 5 of the study and went away without any intervention by the end of the study (Figure S3). STAR particles continued to be applied to the skin every day during that time. It is unclear what role the STAR particles played in the creation and resolution of the rash.

#### 3.3.3 Acceptability

Subjects were again asked to report sensations that they felt during the study. On Day 1, STAR particle application was reported as comfortable by five of the eight subjects (63%), and on Day 10 STAR particles were reported as comfortable by seven out of eight subjects (88%). It is notable that subjects became more comfortable with STAR particles over the course of the study. This increased comfort level is further supported by the scores reported in the variable‐pressure study, where five of six subjects (83%) reported STAR particles as comfortable. Although presented first in this article, the variable‐pressure study was conducted after the repeat‐application study, meaning that the sensation scoring reported in the variable‐pressure study was from subjects with prior STAR particle experience.

**FIGURE 6:**
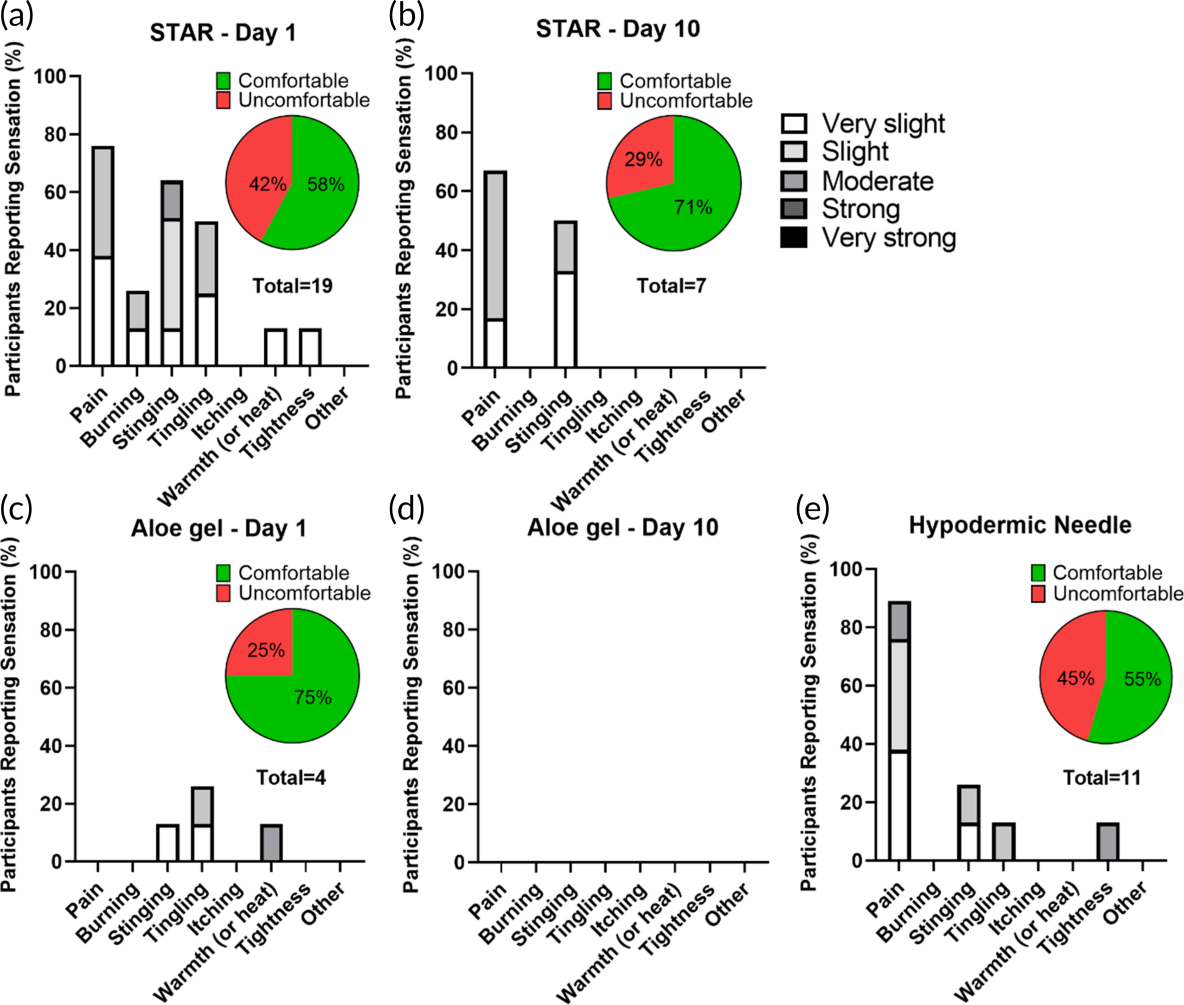
Sensations reported after daily application of STAR particles in human subjects for 10 consecutive days. Frequency that different types of sensation were reported from: STAR particle application at high pressure (80 kPa) on Day 1 (a) and Day 10 (b) of the study, aloe gel application (without STAR particles) at high pressure (80 kPa) on Day 1 (c) and Day 10 (d) of the study, and hypodermic needle application on Day 1 (e) of the study. Insets show percentage of sensations reported to be comfortable. The key on the upper right shows degree of sensation reported by participants. Data were collected from eight human subjects.

Considering the types of sensations, 19 sensations were reported with 11 of them (58%) being described as comfortable on Day 1 of STAR particle application (Figure 6a). The sensations were most frequently reported as pain (32%), stinging (26%) and tingling (21%). By Day 10, the number of sensations dropped to 7, with 5 of them (71%) reported as comfortable (Figure 6b). Subjects described the Day 10 sensations as pain (57%) or stinging (43%). The sensations were generally reported as very slight or slight on both Days 1 and 10. These data further indicate increased familiarity and comfort with STAR particles after repeated use.

For gel application without STAR particles, one subject (13%) found it uncomfortable on Day 1 with a total of 4 sensations reported (Figure 6c). On Day 10, there were no sensations reported during gel application (Figure 6d). For hypodermic needle insertion on Day 1, three subjects (38%) reported discomfort, which corresponded to a total of 11 reported sensations, mostly pain (64%) and stinging (18%), of which 5 (45%) were uncomfortable (Figure 6e).

**FIGURE 7:**
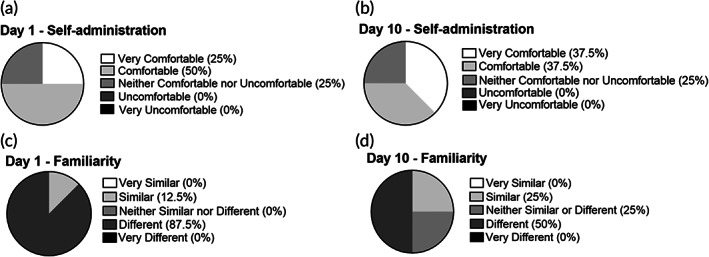
Comfort with self‐administration and familiarity with the experience of STAR particle application in human subjects on Day 1 and Day 10 of the study. Subjects were asked “How comfortable or uncomfortable would you feel applying these skin products by yourself?” on Day 1 (a) and Day 10 (b) of the study. Subjects were asked “How did the application of today's skin products compare to your usual experiences with other skin products (e.g., body lotion, sunscreen, facewash)?” on Day 1 (c) and Day 10 (d) of the study. Data were collected from eight human subjects.

As a further assessment of STAR particle acceptability, 6 (75%) of the subjects reported that they would be comfortable self‐administering STAR particles on Day 1 and Day 10, with slightly greater comfort reported on Day 10 (Figure 7a,b). The remaining two subjects (25%) reported that they would be neither comfortable nor uncomfortable with self‐administration. No one reported being uncomfortable with self‐administration. It should be noted that in this study STAR particles were always administered by study staff, so subjects had not had direct experience with self‐administration.

When asked to compare their experience with STAR particles to their prior experience with commercially available skin products, seven subjects (88%) perceived STAR particles to be different from commercially available skin products on Day 1, but only four subjects (50%) reported a difference on Day 10 of the study, indicating increased familiarity with STAR particles as the study progressed (Figure 7c,d).

Subjects were also asked in an open‐ended manner how we might improve the experience of STAR particle application. Notable feedback was that some subjects would prefer if the skin feel of the STAR particle gel were less grainy and coarse.

## 4 DISCUSSION

This study provides the first data on the effects of varying pressure and repeat application of STAR particles to human subjects and is only the second published study on STAR particles applied to human subjects in any context. Consistent with prior work, this study showed that STAR particles created micropores in the skin and were well tolerated and accepted by the subjects in this study. These findings provide additional support for the translation of STAR particle technology to enable topical delivery of dermatological therapies using a simple, rub‐on formulation.

STAR particles can be used in two ways to improve topical delivery of dermatological therapies. First, STARs can be used as a pre‐treatment before applying a topical product. In this case, STARs would be applied and removed before topical product administration. Alternatively, STAR particles could be incorporated into a topical drug formulation and applied to the skin concurrently with the drug. In this case, the topical formulation (i.e., with the STAR particles) would be left on the skin. They might remain on the skin or fall off over time as the formulation dries.

Because drug absorption with STAR particles is a physical process that relies on the size and number of micropores created in the skin, it may reduce variability in drug delivery compared to conventional topical delivery that relies on patient's intrinsic stratum corneum permeability. However, STAR particles may also introduce variability, as drug delivery is expected to depend on pressure and time of STAR particle application, among other variables.

We found that increasing application pressure caused more microporation of the skin. This suggests that every encounter between a STAR particle microprojection and the skin does not necessarily result in puncture, such that increasing pressure increases the likelihood of a given encounter leading to microprojection penetration into the skin or leading to creation of a larger micropore in the skin. Skin microporation by STAR particles did not vary over the course of repeated application, indicating that the skin was not made more or less susceptible to microprojection penetration over time. Although we did not measure micropore lifetime in this study, we expect the micropores to close within a few hours, based on prior studies using microneedle patches.^29^

The dependence of skin microporation on pressure may be helpful in situations where titration of dosing is desirable, thereby enabling patients to customize the amount of drug delivered simply by varying pressure while rubbing on the skin. However, this finding may offer challenges when drug delivery is intended to be consistent and any patient‐to‐patient and day‐to‐day variability of applied pressure could result in variable drug dosing. This variability could be minimized by patient training, use of an applicator that applies a consistent force, modification of STAR particle properties to reduce dependence of skin penetration on force, or other strategies.

Erythema was generally mild, contained within the area of STAR particle application and disappeared within hours. While degree of erythema depended on application pressure, it did not meaningfully change in intensity or area over the course of repeated application for 10 days. We decided to repeatedly apply with the highest application pressure of 80 kPa to investigate the safety, tolerability, and acceptability in the most consequential application scenario, and, even under these conditions, STAR particle treatment was well tolerated and well accepted.

There was one notable adverse event in the form of mild skin irritation that resolved within a few days. Based on input from a dermatological consultant, we hypothesize that it was caused by a reaction to components of the aloe gel that were absorbed into the skin with greater efficiency after STAR particle application. Further research is needed. We did not observe any STAR particles remaining in the skin or breaking off in the skin duration application. STAR particles were thoroughly wiped away after each application to ensure no residual particles on the skin.

Acceptability of STAR particles was high, with all subjects reporting feeling sensation(s) during application, and most subjects finding them to be comfortable. Subjects were also generally comfortable with the idea of self‐administration. The various measures of acceptability—including sensation/comfort, self‐administrability, and familiarity—increase from Day 1 to Day 10 of the repeat‐application study, indicating a positive experience with STAR particles that improved perception of the technology over time.

A strength of this study is that it was performed in human subjects. However, a limitation is that there were only eight subjects in the study, representing a small demographic and limiting the ability to have statistically powered study design. The study was also done in healthy young adults, meaning that information about effects of STAR particles on diseased skin such as hyperkeratotic skin in patients with psoriasis and ichthyosis (i.e., of relevance to dermatological treatments) and aged skin is lacking and needs further investigation.

The study was also limited to 10 days of application which may not be sufficient for some topical treatment regimens that require prolonged topical dosing. This study also applied STAR particle formulations to the forearm for ease of study; however, other anatomic sites (e.g., hands, elbows, face) would provide additional information about the effects of STAR particle‐containing formulations on skin. Although aloe gel used in this study as the vehicle for STAR particle application is claimed to have soothing effects on the skin and thereby might have affected the skin's response, the exposure of the skin to the gel was brief (i.e., ~30 s), during which time very little aloe should be absorbed into the skin.

While skin was examined and erythema was characterized, a more detailed assessment of safety and tolerability of STAR particles is needed. The impact of different STAR particle designs, other formulation compositions, variability caused by patient‐to‐patient differences such as skin condition and self‐administration, and the presence of dermatological drugs should also be investigated.

## 5 CONCLUSION

STAR particles have been shown to increase topical drug delivery to the skin, which holds promise for future dermatological therapies using drugs that currently have poor topical bioavailability.^27^ In this study, we found that varying pressure during STAR particle application to human subjects increased the degree of skin microporation (i.e., corresponding to increased skin permeability), increased skin erythema and increased the intensity of reported sensation during STAR particle application. However, erythema was generally mild, localized to the site of STAR particle application and transient within about 2 h, and sensation was generally reported as comfortable. Repeated application of STAR particles for 10 consecutive days was well tolerated, with no meaningful change in skin microporation or erythema over the course of the study. Acceptability parameters increased from Day 1 to Day 10 of the study, where reported sensation decreased, comfort increased, perceived self‐administrability increased and familiarity with STAR particles increased. Overall, this study suggests that STAR particles can be used to increase topical delivery to the skin with good tolerability, acceptability and repeatability, even when administered with high‐pressure rubbing and after repeated application.

## AUTHOR CONTRIBUTIONS

**Youngeun Kim:** Data curation (equal); formal analysis (equal); investigation (equal); validation (equal); visualization (equal); writing – original draft (equal). **Jae Hwan Jung:** Methodology (equal); resources (equal). **Andrew R. Tadros:** Conceptualization (equal); methodology (equal). **Mark R. Prausnitz:** Conceptualization (equal); funding acquisition (equal); supervision (equal); writing – review and editing (equal).

## CONFLICT OF INTEREST STATEMENT

This conflict of interest has been disclosed and is managed by Georgia Institute of Technology.

### PEER REVIEW

The peer review history for this article is available at https://www.webofscience.com/api/gateway/wos/peer-review/10.1002/btm2.10524.

## Supporting information


**Data S1:** Supporting Information

## Data Availability

The data that support the findings of this study are available from the corresponding author upon reasonable request.
