# Supplementary material for: Tolerability, acceptability, and reproducibility of topical STAR particles in human subjects
Source: Bioeng Transl Med. 2023 Apr 18;8(3):e10524. doi: 10.1002/btm2.10524 (PMC10189432; doi:10.1002/btm2.10524)
Supplement: Supplementary file 1 — Data S1: Supporting Information [file BTM2-8-e10524-s001.docx]

**Supplementary Information**

Tolerability, acceptability, and reproducibility of topical STAR particles in human subjects

Youngeun Kim, Jae Hwan Jung, Andrew R. Tadros, Mark R. Prausnitz

**
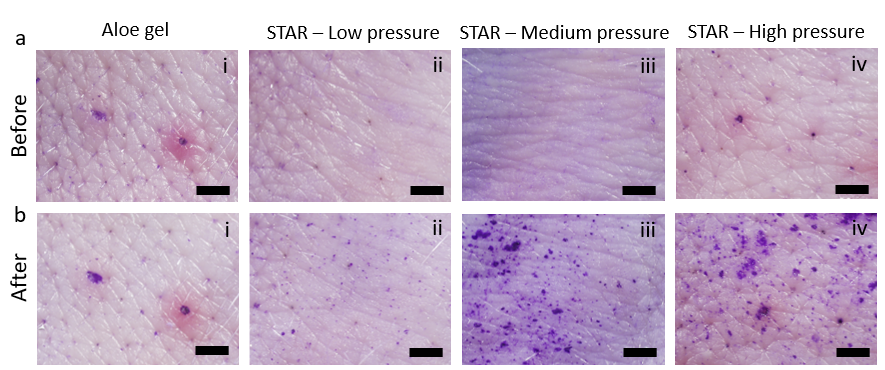
**

**Figure S1.** Micropunctures created by STAR particles on ex vivo porcine ear skin. Representative photographic images of skin before (a) and after (b) applying aloe gel and STAR particles at, low (40 kPa), medium (60 kPa), and high pressure (80 kPa) (scale bar = 2 mm).

**Table S1.** Scoring rubric for assessment of tenderness, erythema and swelling of skin of human participants.


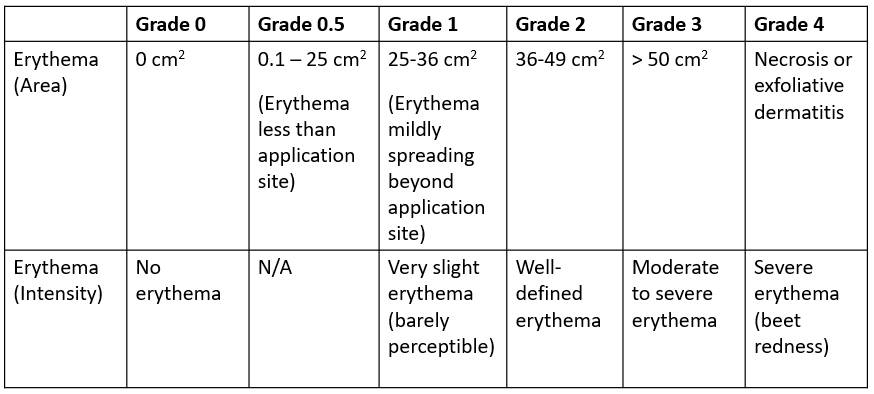


**Table S2.** Demographic information of human subjects the studies of STAR particle application pressure (a) and repeated application of STAR particles (b)


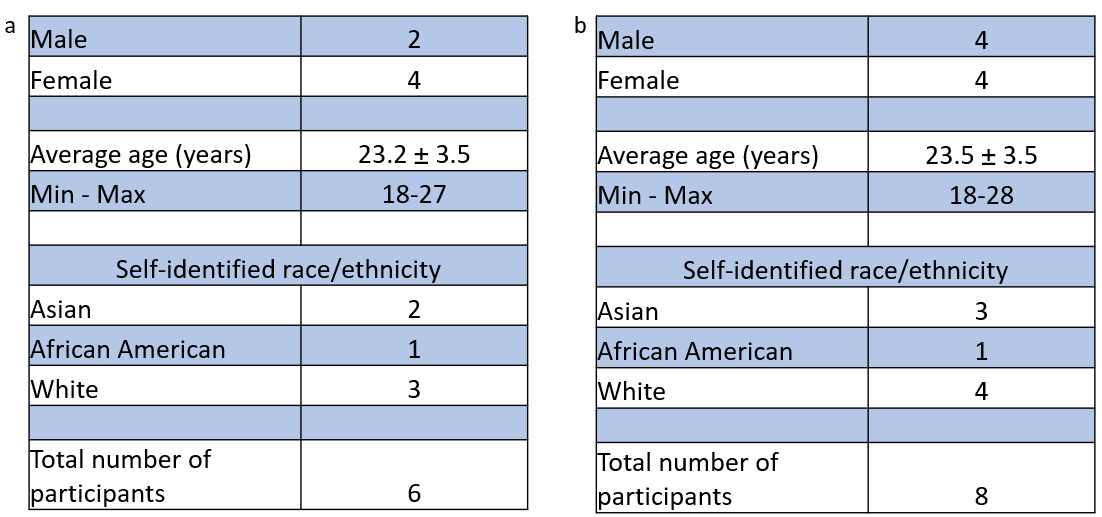


**Table S3.** Analysis of erythema after skin treatments in terms of intensity (a) and area (b) of erythema

**
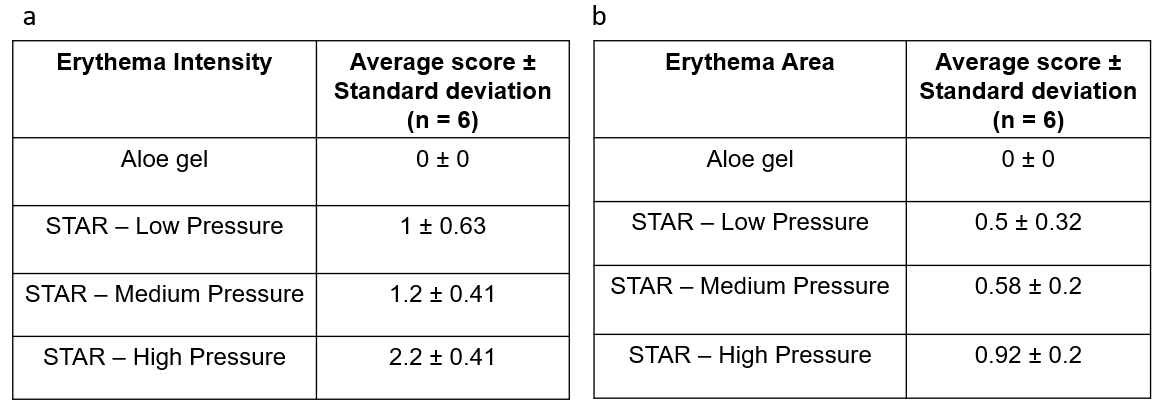
**

**Figure S2.** Correlation between the day of STAR particle application and the erythema disappearance time. Data from each subject was linearly fitted to determine the slope of each fit. The 95% confidence interval of the slope was -0.12 ± 0.2, which included a slope of zero (i.e., there was no significant dependence of erythema disappearance time on day of STAR particle application).


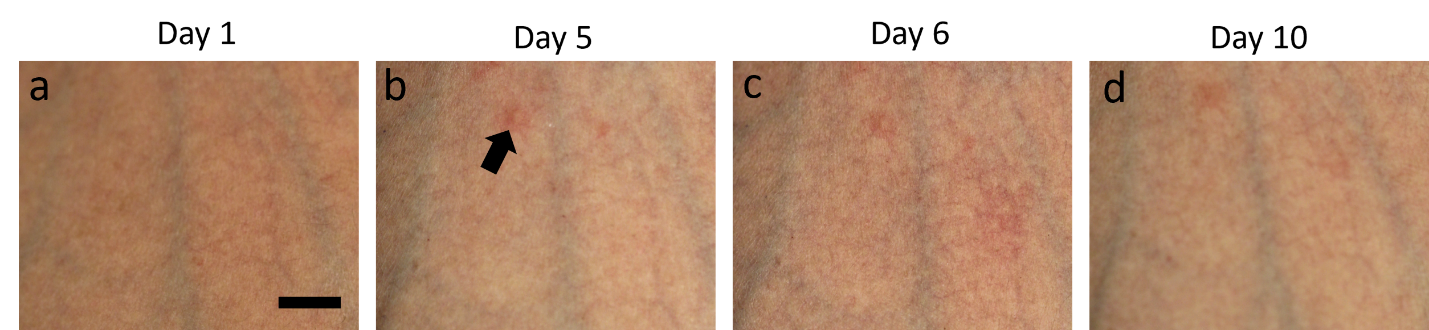


**Figure S3.** Representative photographic images of subject 1’s skin before application of STAR particles on (a) day 1, (b) day 5, (c) day 6, and (d) day 10. The subject had a mild skin irritation that was observed on day 5 of the study (arrow). The irritation went away without any intervention by day 10 (scale bar = 1 cm).
